# Extensive proximal tubular necrosis without recovery following the ingestion of Amanita phalloides: a case report

**DOI:** 10.1007/s40620-021-01018-w

**Published:** 2021-06-18

**Authors:** Andrea Angioi, Matteo Floris, Nicola Lepori, Paola Bianco, Gianfranca Cabiddu, Antonello Pani

**Affiliations:** 1 Division of Nephrology, Dialysis and Transplantation “San Michele” Hospital, ARNAS G. Brotzu, Cagliari, Italy; 2 Pathology Service, “San Michele” Hospital, ARNAS G. Brotzu, Cagliari, Italy; 3 Department of Medical Science and Public Health, University of Cagliari, ARNAS G. Brotzu, Cagliari, Italy

**Keywords:** Amanitin, Amanita phalloides, Intoxication, Acute renal failure, Dialysis, Acute liver failure

## Case description

A 79-year-old Caucasian man and his wife were hospitalized in a peripheral hospital because of nausea, vomiting, watery diarrhea, and oliguria. Symptoms appeared 6 h after ingestion of about 200 g of cooked mushrooms (mistaken by the patient for Amanita caesarea and Boletus impolitus), foraged days before. In the emergency room, the standard protocol for mushroom poisoning was performed (gastric lavage, intravenous acetylcysteine: 150 mg/kg in 60 min, and electrolyte replacement). Twenty-four hours after admission, he showed progressive decline of urine output and an abrupt increase of transaminases. He was therefore referred to our Division the following day, while his wife was discharged after 72 h of clinical observation. His past medical history showed type 2 diabetes with microvascular complications (diabetic retinopathy stage II), and hypertension being treated with angiotensin converting enzyme (ACE) inhibitors.

At admission, his vital parameters were normal (blood pressure [BP] 130/90 mmHg, heart rate [HR] 82/m, body temperature 36.5 °C). Serum creatinine was 7.6 mg/dl, blood urea nitrogen (BUN) 160 mg/dl, with signs of acute liver and pancreas injury (aspartate aminotransferase [AST] 509 U/ml, alanine aminotransferase [ALT] 1,013 U/ml, total bilirubin 2.4 mg/dl, amylase 566 U/l, lipase 1402 U/l, pancreatic amylase 525 U/l). Urinalysis showed normal specific gravity (1.012), glycosuria (50 mg/dl), and mild proteinuria (50 mg/dl), while urinary sediment displayed several granular casts with mild hematuria (16 red cells/HPF). Urinary amatoxin (alpha amanitin) was positive, confirming the diagnosis; interestingly, the toxin was still detected in the urine as late as 14 days after poisoning. Renal ultrasound showed kidneys of normal size, with hyperechoic parenchyma.

**Fig. 1:**
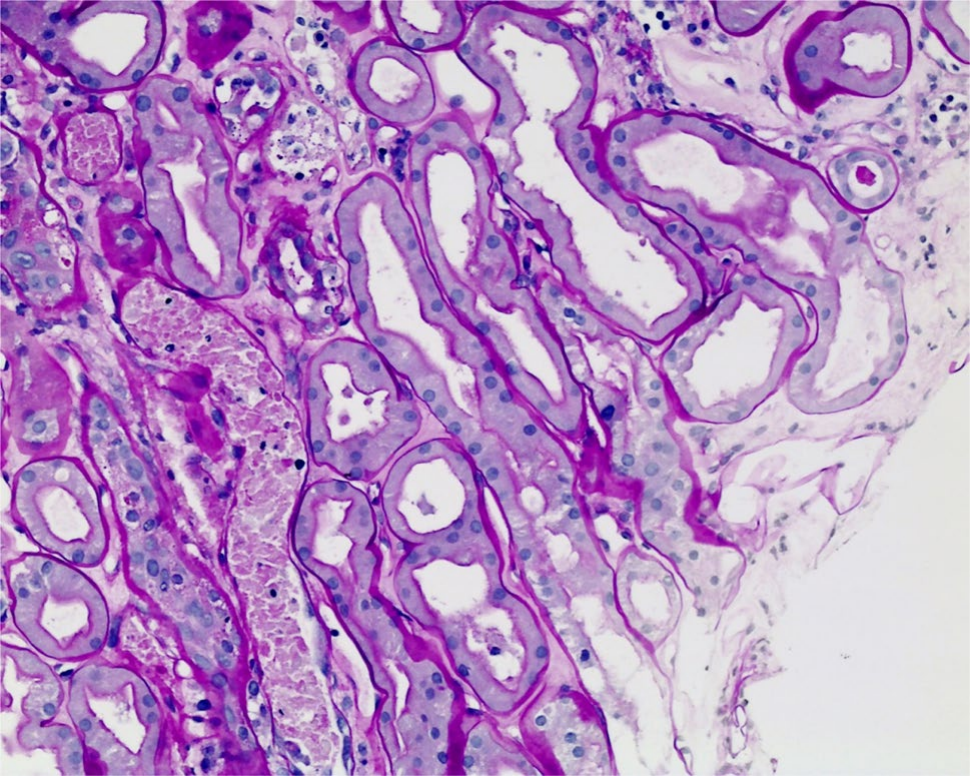
Acute tubular necrosis with granular casts containing nuclear fragments and eosinophilic cytoplasmic debris derived from necrotic tubular epithelial cells (PAS, 100×)

**Fig. 2:**
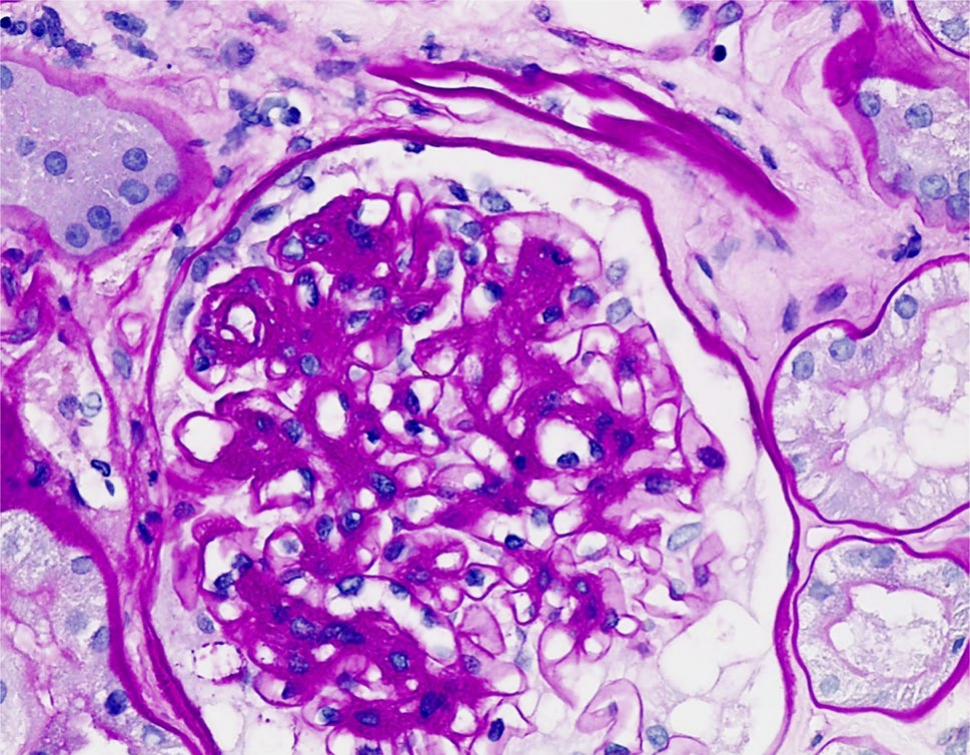
Marked global expansion of the mesangial matrix and cellularity with segmental capillary loop adhesions to Bowman’s capsule. (PAS, 200×)

Intravenous acetylcysteine was continued until his liver parameters improved; dialysis was started with sustained low-efficiency daily diafiltration (SLEDD-f) in an attempt to reduce amatoxin concentration. In view of the lack of kidney function recovery, the patient was later switched to standard hemodialysis. To explore the causes of this persistent kidney failure, we performed a kidney biopsy that showed severe, diffuse, acute tubular necrosis, consistent with the clinical suspicion of amatoxin toxicity superimposed on diabetic nephropathy (Figs. 1 and 2).

The patient was discharged on chronic hemodialysis and five years later he is still undergoing dialysis with no signs of chronic liver disease.

## Lesson for the clinical nephrologist

Amanita phalloides (also called “deathcap”) is a basidiomycete fungus of the genus Amanita known by foragers and clinicians to be a potential cause of fatal acute liver injury. Among food poisoning cases in the US, Amanita phalloides accounts for more than 90% of fatalities, with an estimated mortality of 50% in adults and 33% in children in a dated series [1]. In the absence of up-to-date, high quality evidence, it is legitimate to assume that survival has considerably improved thanks to progress in supportive care and liver transplant [2, 3].

Amanita phalloides produces two types of toxins that cause the phalloideal syndrome: amatoxins and phallotoxins. In humans, phallotoxins exert a direct influence on the cytoskeleton, thus causing a mild and transient enteric syndrome within 6–24 h from ingestion, while amatoxins (in particular alpha amanitin) tightly inhibit RNA polymerase II in the hepatocytes. The impairment of synthetic activity and the loss of structural proteins cause hepatocyte necrosis, which manifests as severe, often fatal, acute liver failure. Kidney failure and anuria are initially the result of dehydration but subsequently kidney damage may be the expression of direct alpha amanitin toxicity to the proximal tubular cells.

As a general rule, once the liver injury is resolved, the renal tubular epithelium progressively regenerates, leading to complete or partial recovery of kidney function [3, 4]. End-stage kidney disease with the need for definitive renal replacement therapy has been described [5]; however, in this condition, renal histology has seldom been reported [6, 7].

Why this patient developed end-stage kidney disease is unclear. Our patient had additional risk factors: type 2 diabetes, hypertension, and a previously known chronic kidney disease. Indeed, although not quantitated, we observed traces of alpha amanitin in the urine 14 days after ingestion, suggesting a relevant toxin load; whether the loss of kidney function is dose-related or independent is not known. It is also reasonable to assume that age, which increases the risk of developing comorbidities, has an impact on the renal and overall outcomes.

In animal models of poisoning by Amanita phalloides, the histological pattern is that of acute tubular necrosis and reactive interstitial nephritis [8]. In our patient, interstitial inflammation was mild, with focal tubulitis and mild interstitial edema, and there were foci of regenerating tubular epithelium, which led us to assume there would be prompt recovery. We were unable to precisely determine the prognosis because (1) how to determine the extent to which tissue has undergone irreversible functional injury after severe acute tubular toxicity is not known; (2) the prognostic value of tissue regeneration on kidney histology is misleading; (3) we do not know what the real proliferating capacity of tubular cells with chronic kidney disease in the background is.

Despite all the measures undertaken to reduce the toxic load as per the current literature data (Table 1), and taking into consideration the pre-existing CKD, prolonged toxicity may explain the dissociation between liver recovery and kidney failure.

**Table 1:** Current literature data

Category	Intervention	Rationale	Dose (or target)	Evidence
Prompt consultation with a regional poison center		Provide the best-known clinical approach in rare toxic syndromes		High
Supportive care	Volume repletion; electrolyte correction; bicarbonate repletion	Avoid hypovolemic shock and reduce the risk of tubular necrosis	Personalized	High
Gastrointestinal decontamination	Gastric Lavage	Remove the contaminated meal and the amatoxin concentration	Gastric emptying, synergic with activated charcoal	High
	Multiple-dose activated charcoal	Absorb amatoxin in the enteric tract to reduce amatoxin concentration	50 g every 4 h or 25 g every 2 h. Some use it days after the meal to interrupt the enterohepatic reabsorption of amatoxin	High
Enhance clearance	Biliary drainage	Interrupt the enterohepatic reabsorption of amatoxin (observed after liver transplant following amatoxin exposure)	Rapid decrease of concentrations after a mean of 4 days from the procedure	Low
	Therapeutic plasma exchange	Removes amatoxins and support the insufficient synthetic activity of the liver	Useful when significant concentrations of amatoxin are present (first 36–48 h). Can be repeated up to 84 h following the meal	Low
	Intermittent and continuous hemodialysis	Useful for removing amatoxins and replacing renal function when needed	Useful when significant concentrations of amatoxin are present (first 36–48 h), otherwise use it if indicated as renal replacement therapy	Low
	Molecular absorbent recirculating system (MARS)	May remove protein-bound substances and water-soluble toxins	Use it as a bridge for liver transplantation	Low
	Fractionated plasma separation and adsorption system (FPSA)	May remove protein-bound substances and water-soluble toxins	Use it as a bridge for liver transplantation	Low
Amatoxin uptake inhibitors	Silibinin dihemisuccinate (intravenous)	Strong inhibitor of Organic Anion Transporting Polypeptide 1B3 (OATP1B3) transporter. Blocks α-amanitin uptake in hepatocytes	Effective when given within 24 h of ingestion. 5 mg/kg intravenously followed by a continuous dose of 20 mg/kg per day for 6 days or until recovery	High
	Silymarin (oral)	Strong inhibitor of Organic Anion Transporting Polypeptide 1B3 (OATP1B3) transporter. Blocks α-amanitin uptake in hepatocytes	Start with 50–100 mg/kg (max 2 g) of oral silymarin every 8 h, then increase to a maximum of 200 mg/kg per dose (maximum single dose; 3 g) if tolerated, for 6 days	Low
	Benzylpenicillin	Strong inhibitor of Organic Anion Transporting Polypeptide 1B3 (OATP1B3) transporter. Blocks α-amanitin uptake in hepatocytes	May be used within 36 h after mushroom ingestion. Suggested 1 MU/kg/day and 0.5 MU/kg/day	High
Antioxidant therapy	N-acetylcysteine	Inactivate free radicals and support glutathione depletion	150 mg/kg intravenously over 15 min followed by 50 mg/kg over 4 h followed by 100 mg/kg over 16 h	Low
	Cimetidine and vitamin C	Antioxidant effects in animal models of amatoxin-containing mushroom poisoning	Cimetidine: 300 mg i.v. every 8 h until clinical improvement; Vitamin C: 3 g i.v. daily until clinical improvement	Low
Experimental therapies	Polymyxin B	Bind RNA Polymerase II preventing α-amanitin from binding	1 mg/kg/day, 4, 8, and 12 h after the meal	Low
	Amatoxin uptake inhibitors	Significantly inhibit amatoxin uptake into liver cells	Not known	Low
	Aucubin	Antioxidant activity in animal models of alpha-amanitin toxicity	Not known	Low
Liver transplantation	Liver replacement therapy		Clinical signs of hepatic injury are moderate to severe	High

*AKI* acute kidney injury

Our case points out the importance of considering mushroom poisoning as a potential cause of acute liver and kidney failure and highlights the fact that, especially in subjects with a pre-existing kidney disease (hypertension, diabetes), renal failure may be irreversible.
